# Retinal Inflammation, Oxidative Stress, and Vascular Impairment Is Ablated in Diabetic Mice Receiving XMD8-92 Treatment

**DOI:** 10.3389/fphar.2021.732630

**Published:** 2021-08-11

**Authors:** Scott J. Howell, Chieh A. Lee, Julia C. Batoki, Thomas E. Zapadka, Sarah I. Lindstrom, Brooklyn E. Taylor, Patricia R. Taylor

**Affiliations:** ^1^ Department of Ophthalmology and Visual Sciences, School of Medicine, Case Western Reserve University, Cleveland, OH, United States; ^2^ Louis Stokes Cleveland VA Medical Center, VA Northeast Ohio Healthcare System, Cleveland, OH, United States

**Keywords:** diabetic retinopathy, ERK5 kinase, XMD8-92, capillary degeneration, retinal inflammation, BRD4

## Abstract

The global number of diabetics continues to rise annually. As diabetes progresses, almost all of Type I and more than half of Type II diabetics develop diabetic retinopathy. Diabetic retinopathy is a microvascular disease of the retina, and is the leading cause of blindness in the working-age population worldwide. With such a significant health impact, new drugs are required to halt the blinding threat posed by this visual disorder. The cause of diabetic retinopathy is multifactorial, and an optimal therapeutic would halt inflammation, cease photoreceptor cell dysfunction, and ablate vascular impairment. XMD8-92 is a small molecule inhibitor that blocks inflammatory activity downstream of ERK5 (extracellular signal-related kinase 5) and BRD4 (bromodomain 4). ERK5 elicits inflammation, is increased in Type II diabetics, and plays a pathologic role in diabetic nephropathy, while BRD4 induces retinal inflammation and plays a role in retinal degeneration. Further, we provide evidence that suggests both pERK5 and BRD4 expression are increased in the retinas of our STZ (streptozotocin)-induced diabetic mice. Taken together, we hypothesized that XMD8-92 would be a good therapeutic candidate for diabetic retinopathy, and tested XMD8-92 in a murine model of diabetic retinopathy. In the current study, we developed an *in vivo* treatment regimen by administering one 100 μL subcutaneous injection of saline containing 20 μM of XMD8-92 weekly, to STZ-induced diabetic mice. XMD8-92 treatments significantly decreased diabetes-mediated retinal inflammation, VEGF production, and oxidative stress. Further, XMD8-92 halted the degradation of ZO-1 (zonula occludens-1), which is a tight junction protein associated with vascular permeability in the retina. Finally, XMD8-92 treatment ablated diabetes-mediated vascular leakage and capillary degeneration, which are the clinical hallmarks of non-proliferative diabetic retinopathy. Taken together, this study provides strong evidence that XMD8-92 could be a potentially novel therapeutic for diabetic retinopathy.

## Introduction

Diabetic retinopathy is a diabetes complication of the retina, and the leading cause of blindness in the working-age population worldwide (Yau et al., 2012; Forouhi and Wareham, 2019). Hyperglycemic spikes, hypoxia, and diabetes-mediated inflammation can initiate neural and retinal cell dysfunction, oxidative stress, vascular endothelial growth factor (VEGF) production, and vascular leakage in the retina; inducing the onset of non-proliferative diabetic retinopathy. This vascular impairment then elicits neovascularization in the retina; progressing retinal pathogenesis to proliferative diabetic retinopathy (Nyengaard et al., 2004; Tang and Kern, 2011; Semeraro et al., 2015). Laser surgeries, steroids, and anti-VEGF treatments are administered during the later proliferative stage of diabetic retinopathy, but still fail to halt the progression of retinopathy in many diabetics (Blinder et al., 2017; Jumper et al., 2018). Further, treatments for non-proliferative diabetic retinopathy are still lacking, even though vision loss can occur in this earlier stage of diabetic retinopathy (Duh et al., 2017). With such a significant health issue, new drug targets are required to stay abreast of the blinding threat posed by this visual disorder.

The cause of diabetic retinopathy is multifactorial, and an optimal therapeutic would halt multiple inflammatory processes, decrease VEGF production, cease neural cell dysfunction, and ablate vascular impairment. Extracellular signal-related kinase 5 (ERK5) is a mitogen-activated protein kinase (MAPK) that elicits the production of multiple inflammatory cytokines and enhances VEGF production in many inflammatory disorders and in diabetes (Kondoh et al., 2006; Wang and Tournier, 2006; Hayashi et al., 2004; Dorado et al., 2008; Chen et al., 2019). ERK5 is the terminal member of a MAPK signaling pathway that can induce inflammation through a series of phosphorylation cascades. ERK5 can be induced to auto-phosphorylate, can be phosphorylated through upstream interactions with MEK5 (mitogen-activated protein kinase 5), or can be phosphorylated at the C-terminal tail through noncanonical pathways. When phosphorylated, pERK5 translocates to the nucleus and can induce gene transcription of growth factors and inflammatory cytokines (Stecca and Rovida, 2019; Kondoh et al., 2006). Previous studies provide evidence that pERK5 is increased in Type II diabetics, enhances diabetes-mediated neural degeneration, and plays a pathologic role in diabetic nephropathy (Suzaki et al., 2004; Chen et al., 2019). In our current studies, we provide evidence that suggests pERK5 is increased in the retina of STZ (streptozotocin)-induced diabetic mice when compared to non-diabetic C57BL/6 mice. In other inflammatory diseases, increased pERK5 activity induced acute phase cytokine production, enhanced VEGF production, and initiated neural degeneration (Finegan et al., 2015; Wilhelmsen et al., 2015; Stecca and Rovida, 2019). In cancer, inhibition of ERK5 halted proliferative tumorigenesis, and enhanced the efficacy of anti-VEGF treatment (Chung et al., 2013). Taken together, we postulated that ERK5 would be an optimal therapeutic target for diabetic retinopathy.

XMD8-92 is a small molecule inhibitor that blocks nuclear translocation of pERK5 and downstream ERK5-dependent inflammation (Yang et al., 2010; Wilhelmsen et al., 2015; Thompson et al., 2017). XMD8-92 is derived from PLK1 (polo-like kinase 1), which can inhibit the acetyl-lysine binding on BRD4 (bromodomain 4) and halt downstream inflammatory activity (Lin et al., 2016). BRD4 is a conserved protein module that is involved in the recognition of acetyl-lysine residues during transcription, and has been previously reported to play a role in retinal and neural cell dysfunction and degeneration (Eskandarpour et al., 2017; Zhao et al., 2017; Eichenbaum, 2020). Also, in our current study we found that BRD4 expression is significantly increased in the retinas of STZ-diabetic mice when compared to non-diabetic C57BL/6 mice. Collectively, we hypothesized that XMD8-92 would be a good therapeutic candidate for diabetic retinopathy, and tested XMD8-92 in STZ-diabetic C57BL/6 mice.

Diabetes-mediated retinal inflammation is one of the first detectable retinal pathologies in this murine model, wherein hyperglycemia induces multiple inflammatory processes, including IL-6, IL-1β, and VEGF production (Semeran et al., 2013; Tonade et al., 2017; Feng et al., 2018). All of these proinflammatory proteins play a pivotal role in vascular impairment of the retina and the onset of non-proliferative diabetic retinopathy (Aiello and Wong, 2000; Tang and Kern et al., 2011; Gupta et al., 2013; Valle et al., 2019). Additionally, ERK5 can induce IL-6, IL-1β, and VEGF production (Wang and Tournier, 2006; Thompson et al., 2017). In this current study, we determined that administering one weekly 100 μL subcutaneous injection of saline containing 20 μM of XMD8-92 was sufficient to ameliorate diabetes-mediated IL-6 and VEGF production, and significantly decreased IL-1β in diabetic mice. Diabetes also enhances oxidative stress in the retina, which is a precursor to the onset of diabetic retinopathy (Du et al., 2013; Sigurdardottir et al., 2019). The XMD8-92 treatment regimen ablated retinal oxidative stress. Previous studies provide evidence that hyperglycemia impairs the expression and distribution of tight junction protein, zonula occludens-1 (ZO-1), in the retinal vasculature causing vascular permeability (Antonetti et al., 1998; Tien et al., 2013). In this current study, we determined that STZ-induced diabetes significantly decreased ZO-1 in the retina but this decrease was halted in XMD8-92 treated diabetic mice. Further, XMD8-92 treatment significantly decreased vascular leakage in the retinas of STZ-diabetic mice. Finally, in early stages of diabetic retinopathy and in this 8 months murine model, retinal endothelial cells and pericytes die, causing acellular and degenerative capillaries (Kern et al., 2010; Liu et al., 2015; Veenstra et al., 2015). However, capillary degeneration was ablated in the diabetic mice that received XMD8-92 treatment, which is a clinical hallmark of non-proliferative diabetic retinopathy. These findings suggest that XMD8-92 could be a potentially novel therapeutic for diabetic retinopathy.

## Materials and Methods

### Streptozotocin (STZ)-Induced Diabetic Mice

CWRU IACUC approved the animal protocols employed in this study. Diabetes was induced in 10-week-old male C57BL/6J mice (Jackson Laboratories, Bar Harbor, ME) by intraperitoneal injections of (STZ) streptozotocin (MPBio Solon, OH) at 60 mg/kg on five consecutive days. Diabetes was defined by 6 h fasted blood glucose concentrations greater than 275 mg/dl that were verified using glucose-dehydrogenase-based strips (Oak Tree Health Las Vegas, NV) 17 days after the last STZ injection (Day 22). Hyperglycemia was quantified by hemoglobin A1c levels at 8, 20, and 30 weeks post-diabetes using the Crystal Chem Mouse A1c kit (Elk Grove Village, IL). Insulin at 0–0.2U (Eli Lilly, Indianapolis, IN) was administered as-needed to maintain body-weight and prevent catabolic state. Body weight and hemoglobin A1c quantifications are shown in Figure 2 and Table 1.

**TABLE 1:** Clinical Data of Non-Diabetic (ND) and Diabetic (DB) Mice.

Group	Body Weight (g) (Week 8)	Body Weight (g) (Week 30)	%HbA1c (Week 8)	%HbA1c (Week 30)
C57BL/6-ND	33 + 2	39 + 5	5.5 + 0.9	5.1 + 0.9
C57BL/6-DB	26 + 2^a^	30 + 2	12.7 + 2.0^a^	11.7 + 2.2^a^
+XMD8-92-ND	32 + 2	38 + 3	5.3 + 0.9	4.9 + 0.8
+XMD8-92-DB	26 + 2^a^	30 + 2	12.6 + 2.1^a^	11.2 + 2.1^a^

Data are mean ± SD.

a = *p* < 0.01 diabetic (DB) compared to non-diabetic (ND) per group.

Retinal inflammation analyses were performed 2 months, oxidative stress analyses were performed 2 and 8 months, while vascular leakage and capillary degeneration were performed 8 months after diabetic conditions were confirmed. As previously described, these are the optimal time points for these analyses in this murine model (Kern et al., 2010; Veenstra et al., 2015; Lindstrom et al., 2019; Zapadka et al., 2020).

### XMD8-92 Treatment Regimen

XMD8-92 {2-[[2-Ethoxy-4-(4-hydroxy-1-piperidinyl)-5,11-dihydro-5,11-dimethyl-6H-pyrimido[4,5-b] [1,4]benzodiazepin-6-one} was purchased from Tocris Biotechne (Minneapolis, MN). XMD8-92 is a small molecule inhibitor that binds to the DCAML2, PLK4, and TNK1 portion of ERK5, which inhibits the translocation of pERK5 to the nucleus and the induction of ERK5-dependent inflammation. While XMD8-92 controls the biological activity of pERK5, it also interacts with the acetyl-lysine binding portion of BRD4 interrupting BRD4-dependent production of inflammatory cytokines and growth factors. In *ex vivo* cellular models, treatments of 5–20 µM of XMD8-92 inhibited the production of proinflammatory cytokines and growth factors (Finegan et al., 2015; Wilhelmsen et al., 2015; Thompson et al., 2017). To distinguish the proper *in vivo* regimen, subcutaneous injections of 100 μL of saline containing 5, 10, or 20 µM of XMD8-92 were administered twice per week, once per week, or once every other week. We hypothesized that there would be a dose-dependent response to these varying concentrations of XMD8-92 treatments. To statistically test this hypothesis, data were analyzed using one-way factorial ANOVA. This was then validated by analyzing the data a second time using an unpaired t-test with Tukey’s post-hoc analysis, which tests each data point in a random order. As shown in Figures 2, 3, it was determined that the optimal XMD8-92 treatment regimen was 20 µM once per week. Therefore, this treatment regimen began one week after diabetes was confirmed (Day 29) in both diabetic and non-diabetic toxicity controls, and weekly injections of saline containing 20 μM (10 mg/kg) of XMD8-92 was used as the treatment regimen throughout the rest of this study. Mice analyzed at the 2 months time point received seven injections, while mice analyzed at the 8 months time point received 28 injections.

Toxicity parameters of XMD8-92 in this model were defined by weekly body weight measures, lethargy, mortality rate, respiratory stress, and autopsy organ appearance, wherein no toxicity was observed in any of the XMD8-92 treated mice. However, body weight is significantly lower in STZ-induced diabetic mice; yet a healthy body weight is maintained (Liu et al., 2015; Zapadka et al., 2021). For toxicity purposes, body weight differences between XMD8-92 treated non-diabetic mice were compared to untreated non-diabetic mice, and XMD8-92 treated diabetic mice to untreated diabetic mice. To determine statistical differences in body weight and Hemoglobin A1c, a two-way ANOVA was performed using Prism (GraphPad Software, San Diego, CA); wherein the nested groups were diabetes and treatment. We further analyzed statistical differences between non-diabetics and diabetics using an unpaired t-test with Tukey’s post-hoc analysis.

### Isolation of Retinas From Mice

Retinas were isolated as previously described (Veenstra et al., 2015). Briefly, curved forceps were used to apply pressure to the temporal side of the orbit to proptose the eye, allowing the forceps to straddle the globe until it clamped the optic nerve. The eye was enucleated, placed on wax, and the anterior portion of the eye was cut off with a Teflon-coated razor blade at the cornea-sclera junction. The retina was then isolated using a retinal spatula on the posterior eye-cup. Retinas were used in assays detailed below.

### Western Immunoblot Analysis of pERK5 in Mouse Retina

**FIGURE 1:**
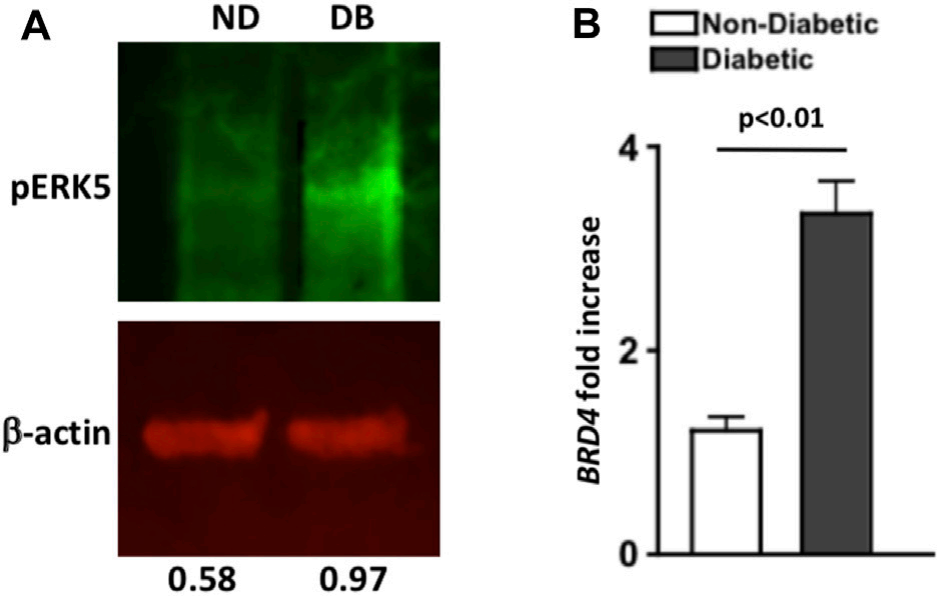
Levels of pERK5 and BRD4 in the retinas of non-diabetic and STZ-diabetic mice. **(A)** Western immunoblot analysis of pERK5 (green bands) in pooled retinal (*n* = 6) lysates of non-diabetic (ND) and diabetic (DB) C57BL/6 mice 8 months after diabetes was confirmed. Red β-actin bands were used as a loading control. Numbers below bands are the level of pERK5 normalized to β-actin using Li-Cor Odyssey Imaging System software. Data are representative of two separate analyses with similar results. **(B)** Fold increase of BRD4 mRNA expression in six pooled retinas of non-diabetic (white) and diabetic (black) C57BL/6 mice; 8 months after diabetes was confirmed. Fold-increase was calculated using the 2 ^ΔΔCT score of each sample (*n* = 3 samples/group). Error bars are the ±SEM, and the *p*-value (*p* < 0.01) was calculated using a student’s unpaired t-test.

Mouse retinas were collected as previously described (Veenstra et al., 2015). Briefly, six retinas were pooled from three non-diabetic or diabetic mice, 8 months after diabetes was confirmed. Retinas (*n* = 6/group) were homogenized in RIPA buffer containing protease/phosphatase inhibitor cocktail (Thermo Fisher, Rockland, IL). Samples were loaded on 4–15% mini-protean gradient gels and transferred to PVDF membrane using the *Trans*-blot Turbo system (BioRad, Hercules, CA). Immunoblots were blocked in Intercept blocking buffer (LiCor, Lincoln, Nebraska) for 1 h at room temperature and then overnight at 4°C in blocking buffer containing a 1:1000 dilution of phospho-ERK5 (Thr218/Tyr220) antibody (#3371, Cell Signaling, Danvers, MA). Following washing and secondary antibody application, the blots were imaged on a Li-Cor Odyssey Imaging System using image studio software (LiCor, Lincoln, Nebraska), and are shown in Figure 1A and Supplemental Figure S1. Quantification of phospho-ERK5 was determined by normalizing samples based on the relative amounts of the good housekeeping gene β-actin present in each sample. Anti-β-actin (#8827, Abcam, Cambridge, MA) was also used as a loading gel control. Immunoblot analysis of pERK5 and β-actin was performed twice using separately pooled samples (*n* = 2 samples/group).

### Quantitative PCR Analysis of BRD4 in Mouse Retina

Retinas (*n* = 6/group) were collected and pooled from three non-diabetic or diabetic mice, 8-months after diabetes was confirmed. RNA was isolated using the RNAeasy mini kit according to manufacturer’s directions (Qiagen, Germantown, MD). Samples with an OD260/280 ratio of 2.0 were used to generate cDNA using SuperScript First Strand synthesis system (Invitrogen, Carlsbad, CA), and quantitative PCR was performed using SYBR green (Applied Biosystems, Carlsbad, CA) as the detecting probe with BRD4 primer (Gene Bank Accession number: NM_198094). Actb (Gene Bank Accession number: NM_007393) primer was used as a loading control. Fold increase of mRNA was quantified using the CT scored data to calculate the 2^ΔΔ CT score; shown in Figure 1B. Analysis was performed on three pooled samples, error bars were generated of the ±SEM, and an unpaired t-test was performed to determine statistical differences between the ΔΔCT scores.

### ELISA Analysis

Sera and protein lysates of three individual retinas of three mice/group were collected for ELISA quantification of IL-6, VEGF, and IL-1β. ELISA analysis was performed according to the manufacturer’s directions (R&D Systems, Minneapolis, MN) at the 2 months time point.

### Quantification of Reactive Oxygen Species (ROS)

At the 2 months time point, retinas (*n* = 5/group) were isolated and incubated in Krebs-HEPES buffer (with 5 mmol/L glucose) for 25 min at 37°C in 5% CO^2^. Luminescence was measured using a Promega GLOMAX 20/20 luminometer, 5 min after addition of 0.5 mmol/L of lucigenin, as previously described (Liu et al., 2016; Sigurdardottir et al., 2019) to quantify the level of ROS per retina.

At the 8 months time point, retinas (*n* = 5/group) were isolated, homogenized in ice-cold Tris-HCl buffer (40 mM, pH 7.4) containing 80U/ml of collagenase (Sigma-Aldrich St. Louis, MO). Retinal cells were collected and incubated with 10 μM of H_2_DCFDA (2′,7′-dichlorodihydrofluorescein diacetate) in the dark for 30 min at 37°C. Fluorescein intensity was quantified using a BD Accuri C6 flow cytometer (BD Bioscience Franklin Lakes, NJ).

### Flow Cytometry of Tight Junction Proteins in the Retina

Individual retinas were digested in the Worthington Papain Dissociation System as per the manufacturer’s instructions (Worthington Lakewood, NJ), and then incubated for 2 h at 37°C in 80U/ml of collagenase (Sigma-Aldrich St. Louis, MO) to isolate retina cells (*n* = 5 retinas/group). Cells were incubated with anti-mouse CD16/32 (Fc block; Thermo Fisher Grand Island, NY) and anti-mouse ZO-1 (ZO-1-1A12; Thermo Fisher Grand Island, NY) in the dark for 30 min at 37°C. Cells were analyzed using a BD Accuri C6 flow cytometer (BD Bioscience Franklin lakes, NJ), gates were set to isotype controls, as previously described (Lindstrom et al., 2019; Sigurdardottir et al., 2019).

### Vascular Leakage in the Retina

Detection of vascular leakage was performed using Evans Blue as previously described (Ou et al., 2019). Briefly, anesthetized mice (*n* = 5/group) received intravenous injections of saline containing 2% Evans Blue, which circulated for 10 min. Mice were then euthanized, and eyes were enucleated and incubated in 4% paraformaldehyde for 2 h. After fixation, retinas were isolated and flat mounts were imaged on a Leica DMI 600B inverted microscope connected to a Retiga EXi camera (Q-imaging Vancouver, BC). The number of leaking vessels was manually quantified, while vascular leakage was quantified by calculating fluorescence intensity in 10 field areas using Metamorph imaging software (Molecular Devices Downington, PA). Representative images of retinal flat mount (upper quadrant) and 100X magnified areas that were quantified (lower quadrant) is displayed in Figure 6A.

### Capillary Degeneration in the Retina

Acellular capillaries were quantified in the retinal vasculature as previously described (Veenstra et al., 2015; Zapadka et al., 2020). Eyes from euthanized mice (n = 5/group) were enucleated and fixed with 10% formalin. After fixation for a 14 day period, retinas were then isolated through a scalpel incision at the cornea-sclera junction. Retinas were incubated in elastase for 2 h at 37°C, followed by an overnight incubation in Tris buffer (pH 8.5). Retinal vasculature was mechanically isolated and stained with hematoxylin and periodic acid-Schiff. Acellular capillaries were manually quantified in seven field areas between the optic nerve and the periphery (200X magnification). Representative images were taken using a 40x objective mounted on an Olympus BX-60 microscope equipped with a Q-imaging Retiga Exi camera. The 10 μM scale bar displayed in Figure 7A is a visual indicator of the size of the representative image.

### Statistical Analysis

Statistical analysis for data displayed in Figures 3–7, was performed first using a one-way analysis of variance (ANOVA) and then an unpaired t-test with Tukey’s post-hoc analysis via Prism (GraphPad Software San Diego, CA). Triplicates of each sample was analyzed, and the mean was equated ±SEM, which is displayed as error bars. A one-way ANOVA was used to determine whether there were any statistically significant differences between the mean of three unrelated groups (non-diabetic, diabetic, and XMD9-92 treated diabetic). We postulated that there would be differences in the non-diabetic vs diabetic samples, but no difference between the non-diabetic vs XMD8-92 treated diabetic samples. Hence, the ANOVA analysis generated a *p*-value and a report that defined statistically significant differences when the *p*-value <0.05. However, ANOVA results do not identify a particular difference between pairs of means when significant. Therefore, we next validated our one-way ANOVA results through the unpaired t-test with Tukey’s post-hoc analysis. This analysis equated the differences between multiple group means, while controlling the experiment-wise error rate.

## Results

### BRD4 and pERK5 Analysis of Retinas From Non-Diabetic and STZ-Diabetic Mice

Levels of pERK5 in pooled retinas (*n* = 6/group) of non-diabetic (ND) and diabetic (DB) C57BL/6 mice was examined through Western immunoblot analysis, 8 months after diabetes was confirmed. Per fluorescence quantification of pERK5 band (normalized to β-actin), there was a higher level of pERK5 detected in the retina of diabetic (0.97) than non-diabetic (0.58) C57BL/6 mice (Figure 1A). A similar trend was detected in a separate immunoblot analysis (Supplemental Figure S1B).

As shown in Figure 1B, BRD4 mRNA expression in pooled retina samples (*n* = 6/sample) was increased ∼3-fold in diabetic (black) than non-diabetic (white) C57BL/6 mice (*n* = 3 samples/group). Per statistical analysis of the ΔΔCT scores, BRD4 expression is significantly increased in the retinas of STZ-diabetic mice; 8 months after diabetes was confirmed. Collectively, these results suggest that pERK5 is enhanced, and BRD4 expression is significantly increased in the retinas of STZ-diabetic mice.

### Clinical Data of *in vivo* XMD8-92 Treatment Regimens in Non-Diabetic and STZ-Diabetic Mice

**FIGURE 2:**
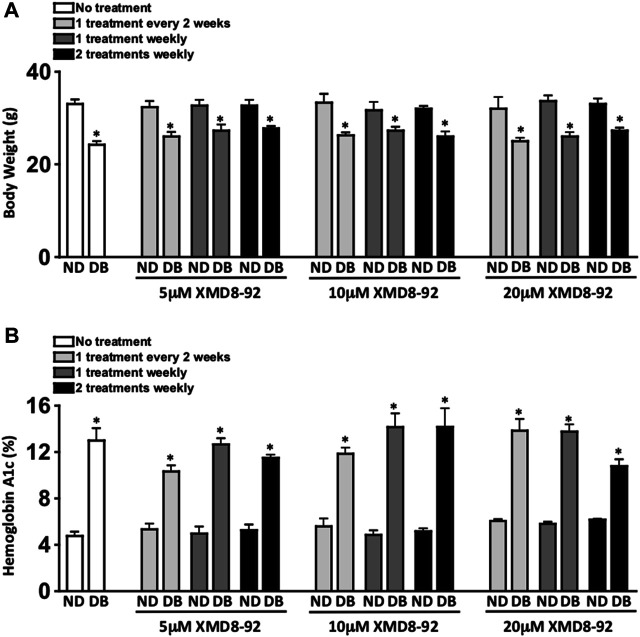
Clinical data of XMD8-92 treatment regimens. Quantifications of body weight **(A)** and hemoglobin A1c **(B)** in non-diabetic (ND) and STZ-diabetic (DB) C57BL/6 mice that received no (white), 5, 10, or 20 μM XMD8-92 treatments. Mice were subcutaneously injected once every other week (light grey), once a week (dark grey), or twice a week (black); 2 months after diabetes was confirmed (*n* = 3/group). Per a two-way ANOVA, wherein groups were nested as treated or diabetic, no significant differences in body weight **(A)** or Hemoglobin A1c **(B)** were found amongst the treated and untreated groups. Per an unpaired student’s t-test a statistically significant difference was calculated as *p* < 0.01 and denotated as * in body weight **(A)** and Hemoglobin A1c **(B)** between the non-diabetic controls and their experimental diabetic counterparts.

To develop an *in vivo* treatment regimen, 100 μL of saline containing 5, 10, or 20 μM of XMD8-92 was administered by subcutaneous injections once every other week (light grey), once a week (dark grey), or twice a week (black). Body weight and hyperglycemia were examined in all of the XMD8-92 treated mice and compared to untreated (white) non-diabetic (ND) and diabetic (DB) controls, 2 months after diabetes was confirmed (Figure 2).

Weekly body weight [measured in grams (g)] of each mouse (*n* = 3/group) was used as one of the toxicity parameters in this treatment model. There was a significant decrease in body weight of all STZ-induced diabetic mice when compared to non-diabetic mice (Figure 2A). However, there was no significant difference in body weight of any of the non-diabetic XMD8-92 treated mice when compared to the untreated non-diabetic mice. Further, there was no significant difference in the body weights of the XMD8-92 treated diabetic mice than the untreated diabetic mice (Figure 2A). Finally, there were no differences observed in lethargy, mortality rate, respiratory stress, or autopsy organ appearance in the XMD8-92 treated than the untreated mice (data not shown). Hence, all of the XMD8-92 treatment regimens tested were not toxic to any of the mice.

Diabetes-mediated hyperglycemia was sustained throughout a 2 month period in STZ-induced diabetic C57BL/6 mice. Sera were evaluated in both untreated and XMD8-92 treated non-diabetic (ND) and STZ-diabetic (DB) mice (*n* = 3/group) to quantify average blood glucose levels through a glycated hemoglobin A1c (HbA_1c_) analysis 8 weeks after diabetes was confirmed (Figure 2B). Both the untreated and XMD8-92 treated diabetic mice had significantly higher A1c blood glucose levels than their non-diabetic controls. However, there was no significant difference amongst the A1c blood glucose levels of the untreated and XMD8-92 treated diabetic C57BL/6 mice (Figure 2B). This suggests that none of these XMD8-92 treatment regimens positively or negatively affect hyperglycemia in this STZ-induced diabetes model.

### Weekly 20 μM Treatments of XMD8-92 Impede Diabetes-Mediated VEGFand Retinal Inflammation

**FIGURE 3:**
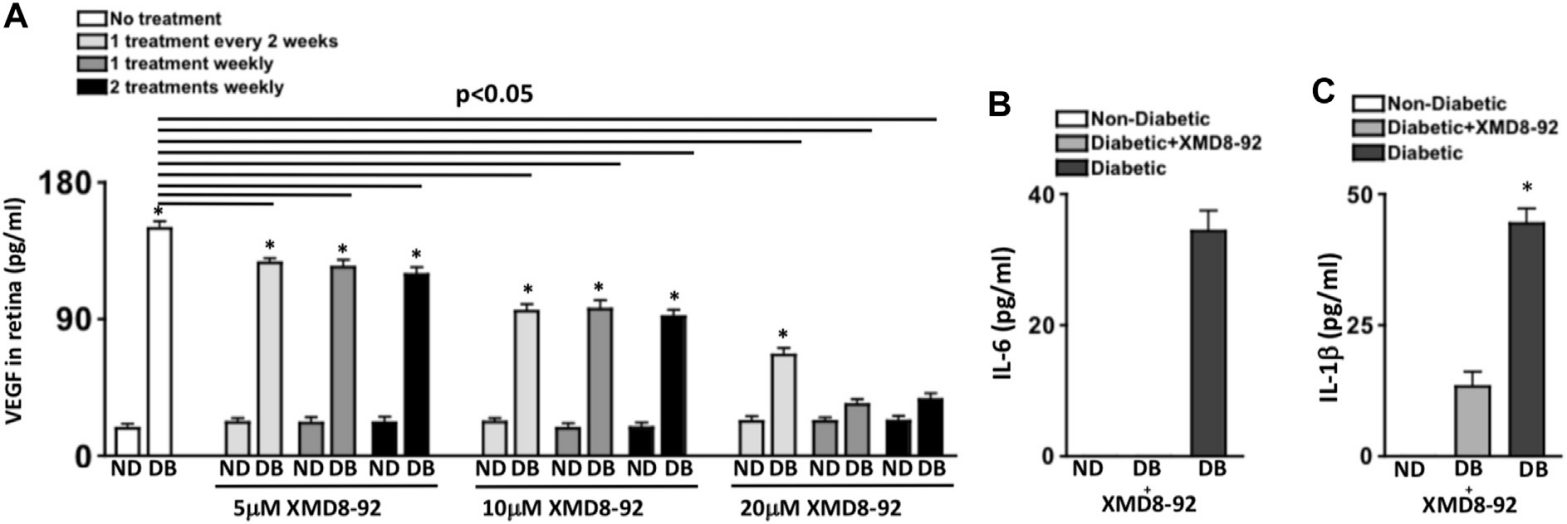
Retinal VEGF and inflammation in diabetic mice receiving XMD8-92 treatment regimens. Quantification of VEGF **(A)** in retinas (*n* = 3/group) of non-diabetic (ND) and STZ-diabetic (DB) mice that received no (white), 5, 10, or 20 μM of XMD8-92 injections 1 time every 2 weeks (light grey), 1 time every week (dark grey), or 2 times every week (black); 2 months after diabetes were confirmed. The horizontal lines represent each XMD8-92 treated diabetic group that is significantly (*p* < 0.05) different than the untreated diabetic group per a one-way factorial ANOVA. Statistically significant differences amongst the non-diabetic and diabetic mice are denoted by an asterisk, wherein * = *p*< 0.01; equated by an unpaired student’s t-test. ELISA quantifications of IL-6 (B) and IL-1β **(C)** in retinas (*n* = 3/group) of non-diabetic (white), 20 μM XMD8-92 treated STZ-diabetic (grey), and untreated STZ-diabetic (black) C57BL/6 mice; 2 months after diabetes was confirmed. * = *p*< 0.01 per an unpaired t-test with Tukey’s post-hoc analysis, wherein all data were analyzed in a random order to equate statistical differences.

To ascertain if any of the XMD8-92 treatment regimens are sufficient to halt diabetes-mediated VEGF, retinas were collected from non-diabetic (ND) and diabetic (DB) mice that received 100 μL of saline containing 5, 10, or 20 μM of XMD8-92 once every other week (light grey), once a week (dark grey), twice a week (black), or were untreated (white), 2 months after diabetes was confirmed (Figure 3).

Protein lysates of individual retinas (*n* = 3/group) were collected for ELISA quantification of VEGF in the retina. As shown in Figure 3A, ∼20 pg/ml of constitutive VEGF was detected in the retina of all non-diabetic mice. The level of VEGF in the retinas of untreated diabetic mice was significantly higher at ∼150 pg/ml, which significantly decreased in XMD8-92 treated diabetic mice in a dose-dependent manner. Levels of VEGF in the retina significantly decreased to ∼120 pg/ml and ∼100 pg/ml in all of the 5μM and 10 μM XMD8-92 treated diabetic mice respectively. Additionally, levels of VEGF in the retina were further reduced to ∼65 pg/ml in the diabetic mice that received 20 μM of XMD8-92 every other week. Finally, the levels of VEGF in the retina of diabetic mice that received either 1 or 2 weekly injections containing 20 μM of XMD8-92 were significantly decreased to ∼20 pg/ml, which is the same level detected in the retinas of the non-diabetic controls (Figure 3A).

**FIGURE 4:**
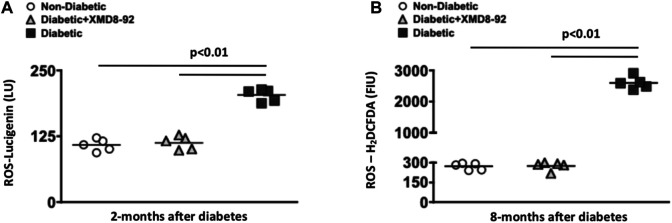
Retinal oxidative stress in diabetic mice. Quantification of reactive oxygen species (ROS) in retinas of non-diabetic (white circles), XMD8-92 treated diabetic (grey triangles), and untreated diabetic (black squares) mice; 2 months **(A)** and 8 months **(B)** after diabetes was confirmed. At the 2 months time point, the level of ROS was detected by quantifying Lucigenin in individual retinas (*n* = 5/group) on a luminometer **(A)**. At the 8 months time point, the level of ROS was detected by quantifying the level of H_2_CFDA in individual retinas (*n* = 5/group) on a flow cytometer. Each data point represents an individual retina. The horizontal lines represent a statistically significant difference (*p* < 0.01) per a one-way factorial ANOVA.

**FIGURE 5:**
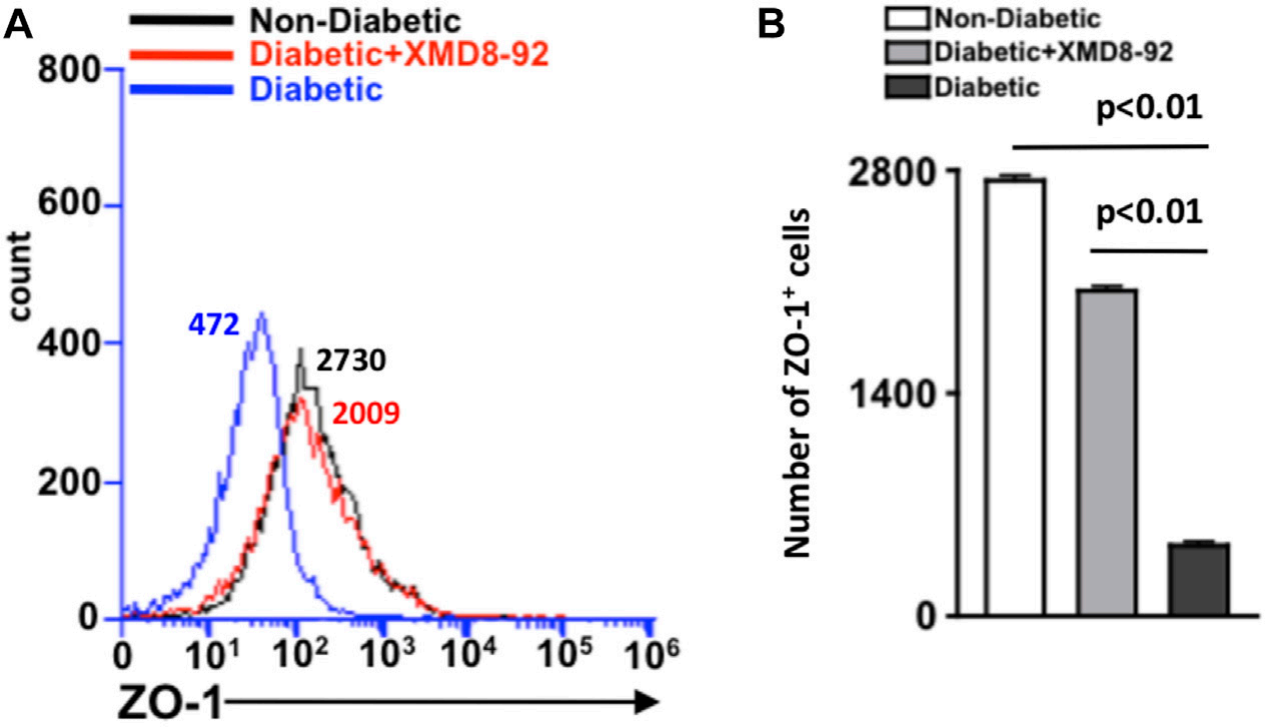
Quantification of ZO-1+ cells in the retina of diabetic mice. **(A)** Representative flow cytometry of the number of ZO-1^+^ cells in the retinas of non-diabetic (black), 20 μM XMD8-92 treated STZ-diabetic (red), and untreated STZ-diabetic (blue) mice, which were gated to an isotype control. Numbers next to overlays indicate the number of ZO-1 positive cells. **(B)** Flow cytometry quantification of ZO-1^+^ cells in the retinas (*n* = 5/group) of non-diabetic (white), XMD8-92 treated diabetic (grey), and diabetic (black) C57BL/6 mice. Error bars represent the ±SEM, and the statistically significant difference of *p* < 0.01 was equated using a one-way ANOVA. The *p*-value and statistically significant difference was further validated by performing an unpaired student’s t-test with Tukey’s post-hoc to equate the *p*-value.

**FIGURE 6:**
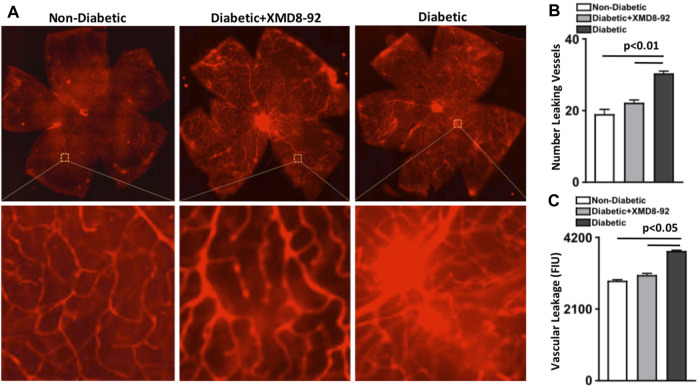
Vascular leakage in the retina of diabetic mice. **(A)** Representative images of vascular leakage in retinas of non-diabetic, 20 μM XMD8-92 treated diabetic, and untreated diabetic mice; following intravenous injections of Evans Blue. Upper quadrant is a full scan of the retinal vasculature, while the lower quadrant displays magnified sections of a leaking vessel. **(B)** Quantifications of the number of leaking vessels in the retinas (*n* = 5/group) of non-diabetic (white), XMD8-92 treated diabetic (grey), and diabetic (black) mice. **(C)** Quantification of vascular leakage measured by fluorescence intensity units (FIU) within a 1.10 μm^2^ area of each retina of all mice. The *p*-value was first equated by determining differences between the three sets of data using a one-way factorial ANOVA, wherein these statistical differences are symbolized by the horizontal lines. Significant differences were then validated and the *p*-value was calculated using an unpaired t-test with Tukey’s post-hoc analysis. All samples were collected 8 months after diabetic conditions were confirmed.

**FIGURE 7:**
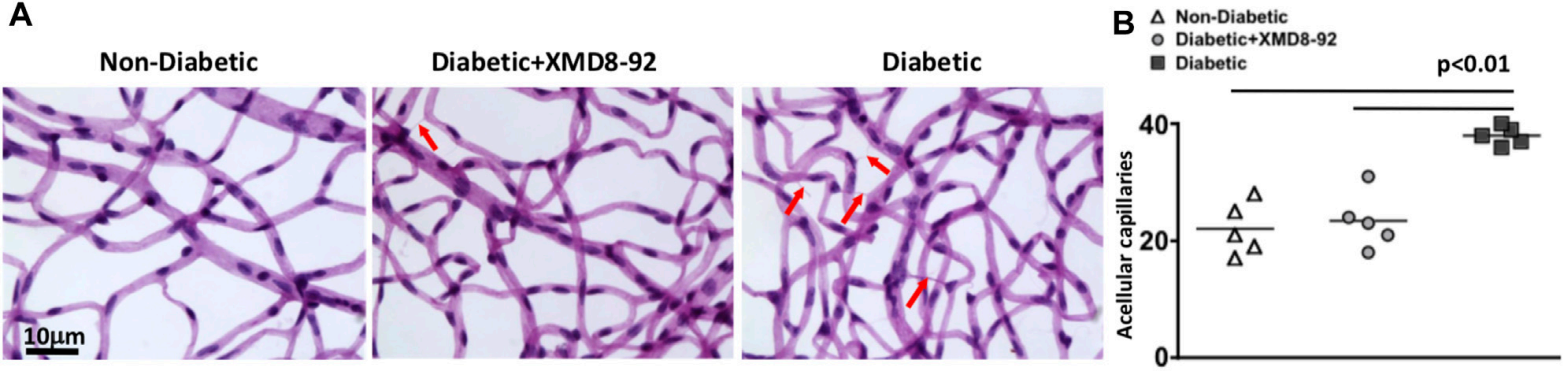
Capillary degeneration in the retinas of diabetic mice. **(A)** Representative images of acellular capillaries in the retinal capillary beds of non-diabetic, 20 μM treated diabetic, and diabetic mice; 8 months after diabetes was confirmed. Scale bars of images = 10 μm. Red arrows highlight acellular images. **(B)** Quantification of acellular capillaries within a 1.10 μm^2^ area of each retina of non-diabetic (white triangles), XMD8-92 treated diabetic (grey circles), and diabetic (black squares) mice. Each data point represents an individual retina from five different mice. Error bars represent the ±SEM, and *p*-values were equated by a one-way factorial ANOVA analysis and further validated by an unpaired t-test with Tukey’s post-hoc analysis.

Taken together, these results suggest that one to two weekly subcutaneous injections of saline containing 20 μM of XMD8-92 is sufficient to halt diabetes-mediated VEGF production in the retina, which is a prevalent precursor to the onset of diabetic retinopathy. Hence, all XMD8-92 treated mice in Figures 4–7 received one weekly subcutaneous injection of 100 μL of saline containing 20 μM of XMD8-92 per week. All clinical data for these mice and the untreated controls are in Table 1.

To evaluate the efficacy of XMD8-92 to inhibit retinal inflammation, retinal protein lysates of untreated non-diabetic (white), XMD8-92 treated diabetic (grey), and untreated diabetic (black) C57BL/6 mice (*n* = 3/group) were collected, and levels of IL-6 and IL-1β in the retina were quantified by ELISA 2 months after diabetic conditions were confirmed (Figures 3B,C **)**. No IL-6 (Figure 3B) or IL-1β (Figure 3C) was detected in the retinas of non-diabetic mice. Conversely, all of these proinflammatory proteins were significantly increased in the retinas of untreated diabetic mice to ∼35 pg/ml of IL-6 (Figure 3B), and ∼45 pg/ml of IL-1β (Figure 3C). Yet these proinflammatory proteins were significantly decreased in the retinas of the XMD8-92 treated diabetic mice, whereas no IL-6 was detected (Figure 3B) and IL-1β was significantly decreased to ∼10 pg/ml (Figure 3C). These results provide evidence that XMD8-92 can sufficiently halt diabetes-mediated retinal inflammation.

### XMD8-92 Treatment Ameliorates Oxidative Stress in STZ-Diabetic Mice

To test the efficacy of the XMD8-92 treatment regimens against diabetes-mediated retinal oxidative stress, reactive oxygen species (ROS) was quantified in the retinas of mice, 2 and 8 months after diabetic conditions were confirmed. At 2 months post-diabetes, levels of ROS were detected using lucigenin in the retinas (*n* = 5/group) of non-diabetic (white circles), 20 μM XMD8-92 treated diabetic (grey triangles), or untreated diabetic (black squares) mice. Levels of ROS was significantly increased in the retinas of untreated diabetic mice when compared to non-diabetic mice (Figure 4A). Yet, levels of ROS were significantly decreased in the mice receiving the 20 μM XMD8-92 treatment regimen when compared to the untreated diabetic mice (Figure 4A). Further, the diabetic mice that receieved 20 μM of XMD8-92 had similar levels of ROS as the non-diabetic controls, with no significant difference amongst these two groups (Figure 4A).

To determine if XMD8-92 can inhibit oxidative stress throughout the progression of diabetes, ROS was quantified in the retinas of mice 8 months after diabetic conditions were confirmed. In this long-term oxidative stress analysis, retina cells were isolated from individual retinas (*n* = 5/group) for H_2_DCFDA detection of ROS levels in the retinas of untreated non-diabetic (white circles), XMD8-92 treated diabetic (grey triangles), and untreated diabetic (black squares) C57BL/6 mice (Figure 4B). Levels of ROS were significantly increased in the retinas of untreated diabetic mice when compared to non-diabetic mice (Figure 4B). Conversely, ROS levels were significantly decreased to levels similar to that found in non-diabetic mice when the diabetic mice received XMD8-92 treatment (Figure 4B). Collectively, these data provide evidence that XMD8-92 can sufficiently inhibit retinal inflammation and oxidative stress.

### XMD8-92 Treatment Halts ZO-1 Degradation in Diabetic Retinas

To examine if XMD8-92 can inhibit the onset of retinal vascular permeability, retina cells were isolated from individual retinas (*n* = 5/group) of non-diabetic (black), XMD8-92 treated diabetic (red), and untreated diabetic (blue) mice, and incubated with anti-mouse ZO-1 antibody for flow cytometry analysis (Figure 5A). As shown in Figure 5B, there is a significant decrease in the number of ZO-1^+^ cells in the retinas of diabetic (black) than non-diabetic (white) mice. However, this decrease is halted when the diabetic mice are treated with XMD8-92, whereas there is no significant difference in the number of ZO-1^+^ cells in the retinas of non-diabetic (white) than XMD8-92 treated diabetic mice (grey). These results provide evidence that XMD8-92 can halt tight junction protein degradation and the onset of vascular permeability in the diabetic retina.

### XMD8-92 Treatment Significantly Decreases Retinal Vascular Leakage in Diabetic Mice

To establish if XMD8-92 can inhibit vascular leakage, Evans Blue was intravenously injected and quantified in retinal whole mounts of non-diabetic (white), XMD8-92 treated diabetic (grey), and untreated diabetic (black) C57BL/6 mice (Figure 6A). Vascular leakage is indicated by diffuse hyper-fluorescence in the retinal vasculature.

First, vessels with hyper-fluorescent leakage were manually quantified to calculate the number of leaking vessels in the retina (*n* = 5/group). As shown in Figure 6B, there was a significantly higher number of leaking vessels in the retina of untreated diabetic than non-diabetic mice, which was significantly lower in the retinas of mice receiving XMD8-92 treatment (Figure 6B). Additionally, there was no significant difference in the number of leaking vessels in the retinal vasculature of XMD8-92 treated diabetic mice than non-diabetic mice (Figure 6B).

Next, vascular leakage in the retina (*n* = 5/group) was quantified by measuring fluorescence intensity using Metamorph imaging software. Similarly, the level of vascular leakage [fluorescence intensity units (FIU)] was significantly increased in the retinal vasculature of untreated diabetic mice compared with non-diabetic mice, which was significantly decreased in the XMD8-92 treated diabetic mice to similar levels measured in non-diabetic mice (Figure 6C). This indicates that XMD8-92 is sufficient to halt vascular leakage in the retina and the onset of non-proliferative diabetic retinopathy.

### XMD8-92 Treatment Ablates Retinal Capillary Degeneration in Diabetic Mice

To determine if XMD8-92 treatment is sufficient to halt capillary degeneration in the retina of diabetic mice, the capillary beds of retinas (*n* = 5/group) were isolated from non-diabetic (white triangles), XMD8-92 treated diabetic (grey circles), and untreated diabetic mice (black squares); 8-months after diabetes was confirmed. All acellular capillaries (representative examples are highlighted by red arrows in Figure 7A) were manually quantified. The number of acellular capillaries in the retinas of untreated diabetic mice was significantly higher than the number detected in non-diabetic C57BL/6 mice (Figure 7B). But the number of acellular capillaries in the retinas of XMD8-92 treated diabetic mice was significantly lower than the untreated diabetic mice, and similar to the non-diabetic mice (Figure 7B **)**. These results provide evidence that XMD8-92 is sufficient to halt diabetes-mediated capillary degeneration in the retinal vasculature. Since this is a clinically relevant hallmark of non-proliferative diabetic retinopathy, we suggest that XMD8-92 would be a good candidate therapeutic for non-proliferative diabetic retinopathy.

## Discussion

Inflammation plays a central role in the onset and progression of diabetic retinopathy (Tang and Kern, 2011; Semeraro et al., 2015). Much of the innate immune receptor signaling that elicits inflammation flows through the ERK5 signaling cascade (Wang and Tournier, 2006; Stecca and Rovida, 2019), which is increased in Type II diabetics (Chen et al., 2019), and our data suggest in the retina of STZ-diabetic mice. Additionally, ERK5 can induce and enhance the production of multiple growths factors; including VEGF (Stecca and Rovida, 2019). Since diabetes-mediated inflammation and VEGF are pivotal precursors to retinal vascular impairment and the progression of diabetic retinopathy (Tang and Kern, 2011; Gupta et al., 2013), ERK5 could be a potentially novel therapeutic target for diabetic retinopathy.

While keeping in mind that an optimal therapeutic for non-proliferative diabetic retinopathy would halt diabetes-mediated inflammation, oxidative stress, and VEGF production, and cease neural cell dysfunction and vascular impairment, we decided to investigate the efficacy of XMD8-92 as a novel therapeutic for non-proliferative diabetic retinopathy. XMD8-92 is a small molecule inhibitor that can cross the blood-retina-barrier, and block pERK5 and BRD4 nuclear translocation and inhibit inflammation (Finegan et al., 2015; Wilhelmsen et al., 2015). XMD8-92 suppressed IL-6 and VEGF production in *ex vivo* inflammation models (Finegan et al., 2015; Thompson et al., 2017). In tumorigenesis models, XMD8-92 hindered vascular angiogenesis and tumor proliferation (Yang et al., 2010; Thompson et al., 2017). Yet the discovery of off target interactions with (bromodomain 4) BRD4 dampened the interest of XMD8-92 becoming a cancer therapeutic (Lin et al., 2016). However, previous ocular studies provide strong support that BRD4 has an important role in photoreceptor cell dysfunction and degeneration (Eskandarpour et al., 2017; Zhao et al., 2017; Eichenbaum, 2020). Further, we discovered that BRD4 expression is increased in the retina of STZ-diabetic mice. Not only is the discovery of enhanced BRD4 in the diabetic retina novel, but it also identifies another potentially novel therapeutic target for diabetic retinopathy. Hence, we postulated that this dual ERK5-BRD4 therapeutic impact would be beneficial in inhibiting the multifactorial causes of diabetes-mediated retinopathy.

Oxidative stress plays a key role in the onset of diabetic retinopathy (Du et al., 2013; Froger et al., 2020). One of the main components of oxidative stress is reactive oxygen species (ROS), which is predominantly produced by photoreceptors in diabetic retinas (Du et al., 2013). We previously reported that diabetes-mediated IL-17A enhanced ROS production in the retina, which was significantly decreased when the constitutively expressed IL-17 receptor was blocked in photoreceptor cells or when IL-17A was systemically ablated (Sigurdardottir et al., 2019). In keratinocytes, when IL-17A binds to the IL-17 receptor, adaptor molecule Act1 can initiate a signaling cascade through TRAF4 (TNF Receptor Associated Factor 4), which can elicit MEK5-dependent phosphorylation of ERK5, which then initiates STEAP4 (Six-Transmembrane Epithelial Antigen Prostate 4) expression (Wu et al., 2015). STEAP4 is a ferrireductase that has a N-terminal oxireductase that binds to NADPH (Nicotinamide Adenine Dinucleotide Phosphate), which can reduce NADPH and initiate NOX (NADPH oxidase) to release ROS (Gauss et al., 2013). In the current study, when one weekly subcutaneous injection of 100 μL of saline containing 20 μM of XMD8-92 was administered throughout 8 months, diabetes-mediated ROS was ablated to similar levels detected in the non-diabetic controls. The same phenomenon was previously detected in the retinas of STZ-induced IL17A^−/-^ diabetic mice (Sigurdardottir et al., 2019). Collectively, it is feasible to suggest that XMD8-92 is ameliorating ROS production in the retina by inhibiting IL-17A-dependent ERK5 activity through this STEAP4 signaling cascade. Mechanistic studies need to be performed to unravel this proposed signaling cascade, which goes beyond the scope of this study and will be a part of our future studies.

Previous studies provide evidence that oxidative stress enhances VEGF production, wherein VEGF is released to protect the retina from further neuronal damage (Froger et al., 2020). Hence it is possible that XMD8-92 treatment is directly ablating diabetes-mediated ROS production, which leads to the VEGF decrease. Alternatively, it is possible that XMD8-92 is directly ablating diabetes-mediated VEGF production, since previous studies provide evidence that ERK5 induces VEGF production in other disease states (Stecca and Rovida, 2019; Thompson et al., 2017). Many mechanistic studies are still needed to elucidate how XMD8-92 ablates diabetes-mediated pathogenesis in the retina. These mechanistic studies will be one of our future focuses. Although the activation mechanism of XMD8-92 is still unclear, we did provide strong evidence that XMD8-92 treatment ablated diabetes-mediated VEGF production in the retina and vascular leakage. Anti-VEGF drugs are one of the few drug treatments for diabetic retinopathy, which is used to treat late stage proliferative diabetic retinopathy or diabetic macular edema (Osaadon et al., 2014; Zhao and Singh, 2018). Yet a significant number of patients do not respond to anti-VEGF treatments for unknown reasons (Zhao et al., 2020). The same anti-VEGF drugs are also used to treat cancer. In previous cancer studies, it was determined that anti-VEGF resistance is driven by IL-17A secreted by tumor resistant cells (Maniati and Hagemann, 2013). While blocking IL-17A and/or ERK5 significantly improved the anti-tumor activity of anti-VEGF, in an unknown mechanistic manner (Chung et al., 2013). Hence, XMD8-92 could block ERK5 and potentially enhance the efficacy of anti-VEGF treatments in non-responders with diabetic retinopathy.

The most prominent finding in this study was the therapeutic impact of XMD8-92 on vascular leakage and capillary degeneration in the diabetic retina. Both vascular leakage and capillary degeneration are well-established as clinically relevant signs of non-proliferative diabetic retinopathy (Bresnick et al., 1977; Yun et al., 2017). Although future mechanistic studies need to be performed to fully delineate the role of ERK5 and BRD4 in the onset of non-proliferative diabetic retinopathy, it is notable that this study identified a potentially novel therapeutic that could ablate diabetes-mediated retinal pathogenesis and vascular impairment. Collectively providing evidence that XMD8-92 is a good candidate therapeutic for non-proliferative diabetic retinopathy.

In conclusion, we developed an *in vivo* XMD8-92 treatment regimen that could significantly decrease diabetes-mediated retinal inflammation, VEGF production, and oxidative stress. We provided evidence that XMD8-92 could halt the degradation of ZO-1, which is a tight junction protein associated with diabetes-mediated vascular permeability in the retina (Tien et al., 2013). Finally, our *in vivo* XMD8-92 treatment regimen ablated diabetes-mediated vascular leakage and capillary degeneration, which are the clinical hallmarks of non-proliferative diabetic retinopathy. Taken together, this study provides strong evidence that XMD8-92 could be a potentially novel therapeutic for the onset and progression of diabetic retinopathy.

## Data Availability

The original contributions presented in the study are included in the article/Supplementary Material, further inquiries can be directed to the corresponding author.
