# Supplementary material for: Retinal Inflammation, Oxidative Stress, and Vascular Impairment Is Ablated in Diabetic Mice Receiving XMD8-92 Treatment
Source: Front Pharmacol. 2021 Aug 11;12:732630. doi: 10.3389/fphar.2021.732630 (PMC8385489; doi:10.3389/fphar.2021.732630)
Supplement: Supplementary file 1 [file DataSheet1.PDF]

Supplemental Figure S1

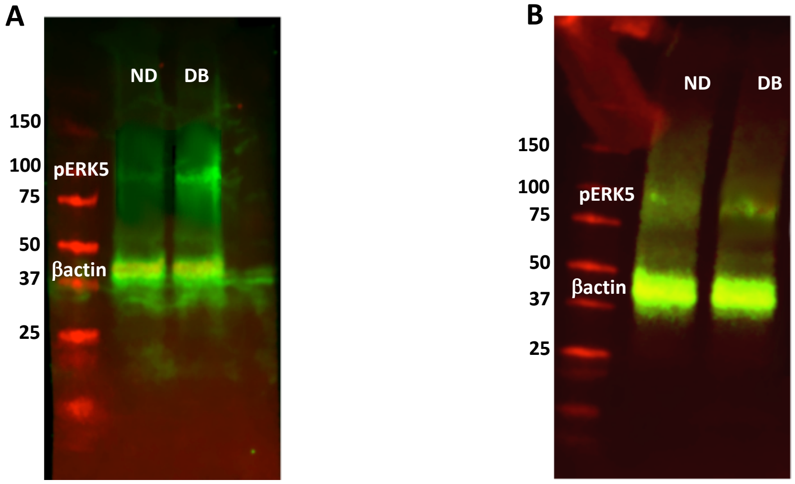

Supplemental Figure S1. Phospho-ERK5 Western immunoblot gels of retinal protein lysates of non-diabetic and STZ-diabetic C57BL/6 mice. Two separate immunoblot analyses of two separate protein lysate samples of 6 pooled retinas of non-diabetic (ND) and diabetic (DB) C57BL/6 mice; 8-months after diabetes was confirmed. Protein lysates were probed with anti-pERK5 (green bands) and anti-β-actin (red bands) as a loading control.

Supplemental Figure S2

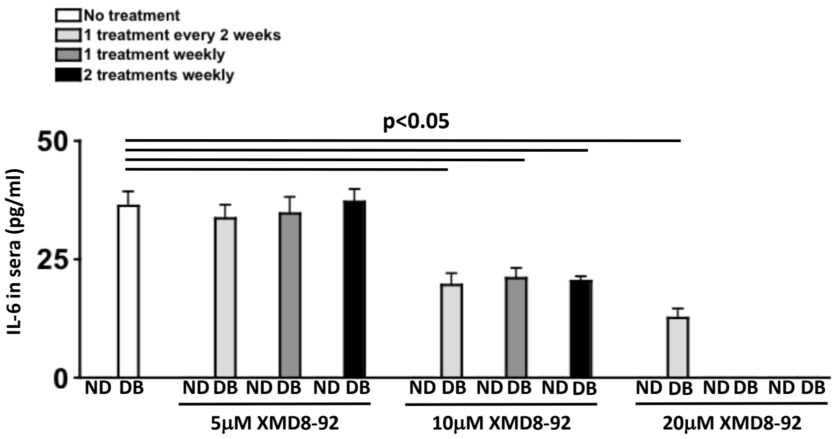

Supplemental Figure S2. IL-6 in the sera of non-diabetic, XMD8-92 diabetic, and untreated diabetic mice. Quantification of sera levels of IL-6 in non-diabetic (ND) and STZ-diabetic (DB) C57BL/6 mice that received no (white), 5µM, 10µM, or 20µM XMD8-92 treatments. Mice were subcutaneously injected once every other week (light grey), once a week (dark grey), or twice a week (black). Sera was collected 2-months after diabetes was confirmed from individual mice (n=3/group). The p-value ($p<0.05$) signifies statistical differences that were equated using a one-way factorial ANOVA, with a hypothesis that there would be a dose-dependent impact of XMD8-92. This statistical finding was then validated using an unpaired t-test with Tukey's post-hoc analysis that analyzed data in a random order. The horizontal lines represent the groups that are significantly different from one another.

Supplemental Figure S3

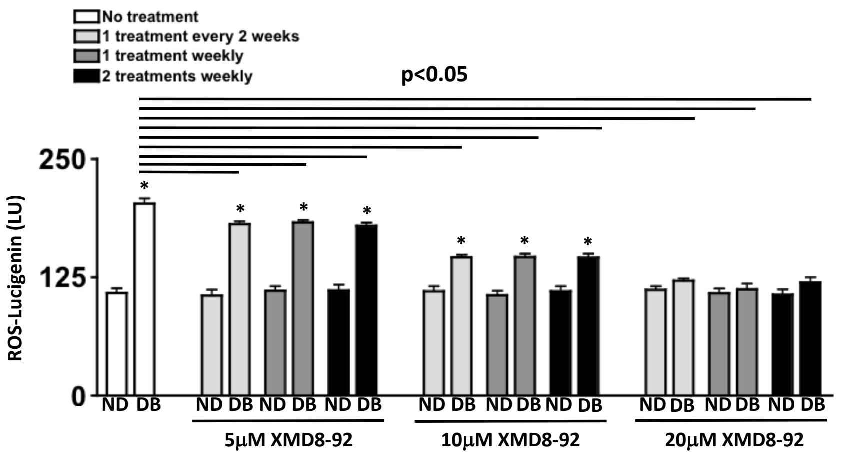

Supplemental Figure S3. Levels of retinal ROS in non-diabetic, XMD8-92 diabetic, and untreated diabetic mice. Quantification of ROS (reactive oxygen species) in individual retinas (n=3/group) of non-diabetic (ND) and STZ-diabetic (DB) C57BL/6 mice that received no (white), 5µM, 10µM, or 20µM of XMD8-92 treatments. Mice were subcutaneously injected once every other week (light grey), once a week (dark grey), or twice a week (black). Individual retinas of 3 separate mice were analyzed 2-months after diabetes was confirmed. The p-value ($p<0.05$) signifies statistical differences that were equated using a two-way factorial ANOVA, with the two-nested factors being diabetes and XMD8-92 treatment. This statistical finding was then validated using an unpaired t-test with Tukey's post-hoc analysis. The horizontal lines represent the groups that are significantly different from one another.
